# Production *Ganoderma lucidum* extract nanoparticles by expansion of supercritical fluid solution and evaluation of the antioxidant ability

**DOI:** 10.1038/s41598-022-13727-8

**Published:** 2022-06-14

**Authors:** Mehrnaz Karimi, Farhad Raofie, Mehrdad Karimi

**Affiliations:** 1 Department of Analytical Chemistry and Pollutants, Shahid Beheshti University, Tehran, 198396941 Iran; 2 Department of Traditional Medicine, School of Persian Medicine, Tehran University of Medical Sciences, Tehran, 1417613151 Iran

**Keywords:** Chemistry, Nanoscience and technology

## Abstract

Due to the growing human tendency to treat with natural substances, fungi such as Ganoderma lucidum can be a good source to meet this need. Effectiveness, ease of use and a rich source of active ingredients such as ganoderic acids have caused *G. lucidum* to be considered in the pharmaceutical and food industries. In this project, *G. lucidum* was applied to extraction using supercritical carbon dioxide. Then expansion of supercritical fluid solution (ESS) was used as, novel, repeatable and green method to yield nanoparticles from *G.lucidum* extract. The response surface method was used to improve the Extraction efficiency, antioxidant activity, and improving the nanoparticles production status. Optimal conditions were observed at the extraction step by setting pressure at 27.5 MPa, dynamic time of 46 min, and modifier volume of 162 μL. The optimum point for the production of nanoparticles was obtained as follows: pressure drop at 25 MPa, 20 min for collection time, and 40° C for temperature. Under these conditions, the size and count were 86.13 nm, and 98, respectively. Nanoparticles were analyzed by FESM and, the DPPH was used for antioxidant activity evaluation. The LC–MS identified various ganoderic acids from *G.lucidum* that are famous to be highly oxygenated triterpenoids.

## Introduction

For two thousand years, mushrooms have been considered healthy food due to their nutrients, pleasant taste, food supplements, and medicinal sources^1^. *Ganoderma lucidum* (Fr.) Krast , which belongs to the family of Ganodermataceae of Polyporales, is a fungus that has been used as popular folk medicine and tonic for promoting longevity for centuries in East Asia^2,3^. It is known as Lingzhi’’ in Chinese, ‘‘Reishi’’ or Mannentake in Japanese and, ‘‘Youngzh’’ in Korean^4^. Identifying the bioactive compounds of medicinal plants can be helpful in understanding the complex activity of herbal medicines. Triterpenes, polysaccharides, sterols, lectins, and some proteins are the major pharmacologically chemical constituents of *G. lucidum* and related species^5^. More than 140 triterpenoids have been identified from different parts of *G. lucidum*^6^. Ganoderic acids, one of the most potent therapeutic biomolecule triterpenes detected in *G. lucidum*, consist of three cyclohexane rings and one cyclopentane ring^7^. These compounds have antioxidant, anti-HIV, antibacterial, anticancer and anti-inflammatory effects^8^. Because ganoderic acid has very small solubility in water, its biological activity is low, which limits its use in industry and medicine. Biosorption problems, which mainly depend on solubility, constitute a significant obstacle to the development of new drugs. Solubility strongly depends on the size, morphology, and particle size distribution^9,10^. So, reducing particle size is one of the main options among different strategies to increase biological accessibility and consequently increases the total surface area. Significant challenges in nanomaterials science control the size, size distribution, morphology, and particle shape, which are directly related to nanomaterial preparation^11,12^. Several traditional methods, such as grinding, crushing sublimation, evaporation, milling, crystal engineering treatment, and etc^13,14^ have been exerted to diminish the particle size, but they have various problems such as low efficiency, long distillation time, loss of volatile components, degeneration of products and etc^15^. Also, some modern methods, such as ultrasonic and microwave, have disadvantages such as the use of large amounts of solvent, solvent retention, and time consuming^16^. The production of nanoparticles of pharmaceutical compounds using supercritical fluid, especially carbon dioxide supercritical fluid (SC-CO_2_)^17,18^, has recently been considered in the pharmaceutical industry. Precise control of the crystallization process is achieved by using supercritical fluid, resulting in favorable conditions for producing very fine, and uniform particles^19^. In this method, the fluid used is in the group of safe solvents and is also an efficient alternative to conventional methods in terms of time and cost used^20^. These properties reduce the environmental impact, and introduce supercritical fluid extraction as an environmentally friendly method^21–23^. Due to the unique properties of the target compounds, the supercritical fluid is used as a solvent (e.g., Rapid expansion of supercritical solutions (RESS)), anti-solvent (e.g., supercritical anti- solvent (SAS)), and intermediate medium (e.g., particles from gas- saturated solution (PGSS)). In the present methodology, nanoparticles are produced based on the expansion of supercritical fluid solution (ESS) method previously introduced by our team^24–29^. ESS is a modified method of RESS that the precipitation process was happened by decreasing the pressure suddenly. In this method, Unlike RESS, the secondary pressure is higher than the supercritical pressure, and the produced particles are smaller in size. In addition, the role of oxidative stress in developing certain diseases has been identified^30^. Synthetic antioxidants or natural antioxidants containing high concentrations of antioxidants such as *G. lucidum* can reduce oxidative damage^31^. So, the relation of antioxidant properties with decreasing particle size was investigated.

Therefore, it can be said that today, due to the importance of natural drug resources and their metabolites in ensuring the health of human societies and their high economic potential, the selection of appropriate methods for extracting effective drugs from natural sources has received more and more attention. Ganoderma, which has been considered by humans for thousands of years, contains compounds that play an important role in human health. Therefore, in this study, the extraction of triterpenoids of this plant, which includes ganoderic acids, was considered. Also, the use of an introduced new method (ESS), which is the outcome of our research team efforts, was used to produce ganoderic acid nanoparticles from *G.lucidum* extract. In this method, the use of a small amount of organic solvent, along with applying supercritical fluid method, causes the least amount of environmental pollution. In addition to the FESEM assay to prove the production of nanoparticles, the antioxidant properties of the produced nanoparticles were also evaluated using the DPPH assay. Further, the production conditions of extraction, nanoparticles and their antioxidant properties were studied to achieve the optimal point by response surface methodology.

## Experimental

### Reagents

Carbon dioxide with purity greater than 99.99% was provided by Roham. co. (Tehran, Iran). HPLC grade methanol, acetonitrile, and dimethyl sulfoxide (DMSO) were prepared from Caledon (Georgetown, Ont., Canada). Analytical grade of ethanol as a solvent Merck, Darmstadt, Germany), distilled and deionized water, and 2,2-Diphenyl-1-picrylhydrazyl, Free Radical (DPPH, Sigma, USA), were applied.

### Preparation of sample

The fruiting bodies of *Ganoderma lucidum* were purchased from the Sarinfam Mushroom Company. The Sarinfam Mushroom Company cultivates mushrooms. The fruiting bodies of G. lucidum have been collected in full compliance with all necessary laws and protocols with the license number of 104134410 from the Ministry of Mining Industry and Trade of Iran. Taxonomic identification was confirmed by Dr. Mohammad Reza Asef Shaian a voucher specimen (No. ASGL 16) is stored at the herbarium of Iranian Research Institute of Plant Protection (Tehran, Iran). The plant material was dried, and then the milling (laboratory mill, Myson, China) and sieving process were accomplished to form a uniform powder plant with a particle size of 0.1 mm or less by standard mesh size sieves. The prepared sample was stored at 3 ± 1° C (Fig S2).

### *Ganoderma Lucidum* extraction procedure

**Figure 1:**
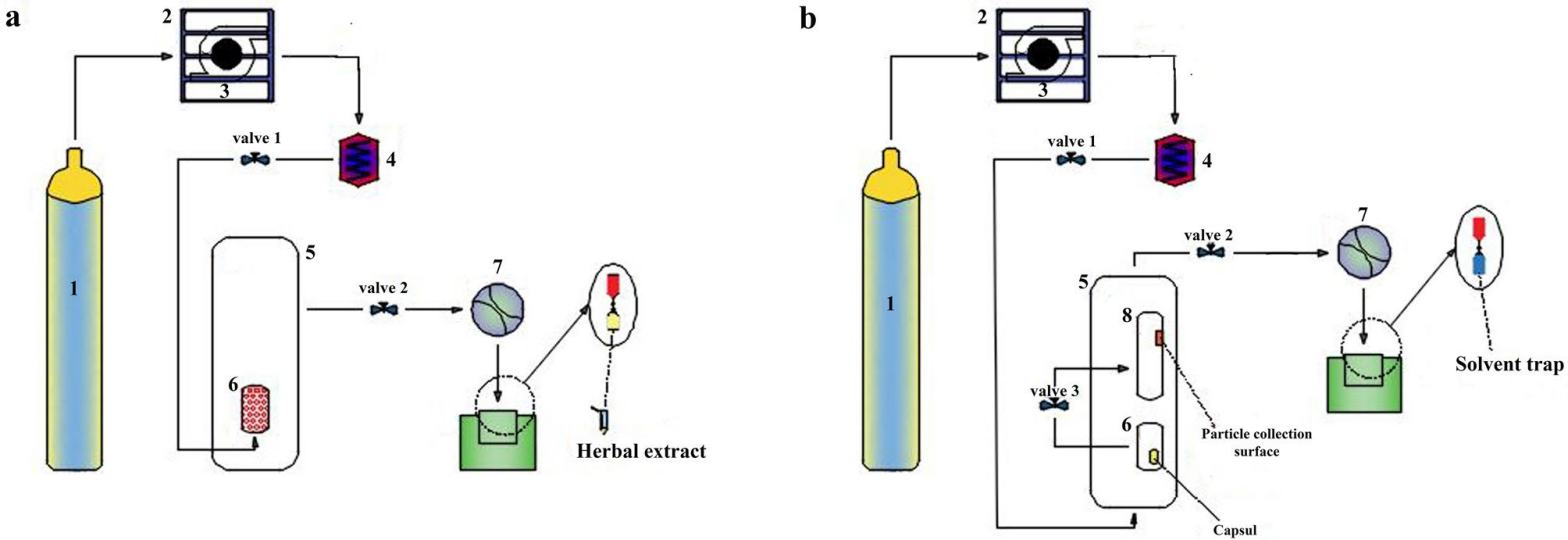
(**a,b**) The schematic diagram of the SFE and ESS set up: (1) CO_2_ cylinder, (2) chiller, (3) high-pressure syringe pump unit, (4) heat exchanger, (5) oven, (6) extraction/equilibration vessel, (7) restrictor, (8) collection vessel.

To obtain *G. lucidum* extract, the environmentally friendly supercritical fluid method was applied. The device used for extraction in this work was Suprex MPS/225(Pittsburg, Virginia, U.S.A.) on a laboratory scale, as shown in Fig. 1a. The device was used in supercritical fluid extraction (SFE) mode with a maximum operating pressure of 40 MPa. For each run, the stainless steel extraction chamber (3 mL volume) was filled with 0.50 g ± 0.01 mg of the powdered *G. lucidum* with the glass bead (1 mm in diameter). Before connecting the chamber to the system, a modifier was poured into it. Two filters, made of porous steel, are placed at both ends of the chamber, so that, only carbon dioxide can pass through it. Carbon dioxide is cooled by a chiller to achieve stable conditions after filling and closing the system. A back pressure regulator and a restrictor (Dura flow manual variable; Suprex Co.) adjust the pressure and output current from the chamber, respectively. A heater with temperature adjustment is used to prevent freezing and obstruction of the restrictor outlet. To improve the extraction efficiency, and to avoid solvent evaporation during collection, a 4.00 mL vial as a collector was put in an ice bath. After each step was completed, the extraction vials were stored at room temperature for evaporation of the solvent to collected the dried extract. The extraction efficiency is calculated as follows (Eq. 1) (W is an abbreviation of weight):

1 $${\text{E}}_{{{\text{extraction}}}} \% = \, \left( {{\text{W}}_{{\text{dried extract}}} /{\text{W}}_{{{\text{sample}}}} } \right) \, \times {1}00$$

Numerous factors such as pressure, temperature, modifier volume, static time, and dynamic time can affect the extraction efficiency of supercritical fluid and the amount of IC_50_ alone or by interaction with each other. In this study, using Statgraphics XVII software, a screening step was performed to find parameters (Table S1) affecting extraction efficiency and IC_50_. Then, using the surface response methodology, the optimal point for the effective parameters was obtained.

#### Selection modifier

Since carbon dioxide is non-polar, a modifier is used to extract the polar compounds of the sample^32^. To select the best solvent, four solvents of ethanol, methanol, acetonitrile, and water were used to extract the active ingredients of *G. lucidum* with high efficiency and the lowest value of IC_50_. At this stage, all parameters remain constant, and the appropriate solvent is identified according to the equation given in the previous section.

### Nanoparticle production process

In this study, the nanoparticle production process was based on expanding supercritical fluid solution (ESS) method, introduced by Raofie et al.

The apparatus consists of two vessels in the production stage of nanoparticles; Equilibration (5 mL) and Precipitation (25 mL) vessels connected with a needle valve 3 (Fig. 1b). A specified volume (V) of the extract solution was collected at the optimum point. It was extracted from the plant via the supercritical fluid that could be penetrate in and out of the capsule by making a small hole in the upper part of the capsule and bending it. Then, the extract solution was transferred to a 0.5 ml polyethylene capsule, and placed in an equilibration chamber. Three pieces of 50 × 20 nm mica sheets (Agar Scientific Ltd.) were placed inside the precipitation vessels to collect nanoparticles on their surfaces. The selected values for the parameters of pressure drop (P_d_), temperature (T), equilibrium time(t_1_), and collection time (t_2_) were based on the initial experimental results and information obtained from previous studies. Also, according to previous studies, the effect of equilibrium time was kept constant for 30 min in all runs of the experiment.

To find the optimal point for the highest number of nanoparticles formed with the smallest size, and maximum antioxidant properties, the parameters of pressure drop, collection time and temperature were examined by Statgraphics XVII software. The Box Benchen method was used to optimize the parameters at three levels.

To start the experiment, initially, valve 1 was opened, and CO_2_ was pumped into the equilibrium vessel to dissolve the active ingredients of *G. lucidum* extract in SC-CO_2_. When the pressure reached the desired programmed value (P_1_), valve 1 was closed. After 30 min (t_1_) has elapsed at T ˚C, valve 3 was opened, and the contents of the equilibration vessel are expanded into the precipitation vessel. At this stage, to adjust the pressure in P_2_, valve 2 was opened (flow rate: 0.1 mL min^−1^) followed by a rapid pressure drop (P_d_). After that, for t_2_ minutes, valve 2 was closed and the precipitation vessel was kept at constant pressure (P_2,_ t_2_) to solute nucleation and nanoparticle production. Finally, valve 2 was opened, and the system was released from the pressure. The mica sheets were transferred to special containers for collection of particle size analysis, identification of compounds, and evaluation of antioxidant properties.

### Evaluation of antioxidant activity

The free radical scavenging activity of different extracts was evaluated by DPPH based on the study by Padhan et al.^33^ with some modifications. Collected particles on the mica sheet and herbal extract, at each stage of the designed experiments, were dissolved in methanol. Various concentrations of 3 mL of the extract solution were mixed with 1 mL methanol solution of DPPH (1 × 10^–4^ mol/lit, 25 °C). Then, the obtained mixtures were shaken vigorously, and kept at room temperature for 30 min in a dark place. Eventually, the absorption of each of the solutions was measured at 517 nm using UV–VIS spectrophotometer against a control contains DPPH and methanol. The inhibition of DPPH percentage for each was obtained by the following equation (Eq. 2):

2 $${\text{R}}_{{\text{S}}} \left( \% \right) = \, \left( {{\text{A}}_{0} {-}{\text{ A}}_{{1}} } \right) \, /{\text{A}}_{0} \times { 1}00\%$$

Here R_S_ is the radical scavenging activity, A_0_ is the absorbance of control solution, which contains all reagents except analyte, and A_1_ is the absorbance of analyte solution. All experiments were done in triplicate, and the mean value was calculated. After that, the IC_50_, which indicated the concentration of the extract solutions that causes scavenging the 50% of DPPH free radicals, was obtained from the inhibition diagram in terms of the concentration of the extract solution and was reported as antioxidant activity. Lower amounts of the IC_50_ indicate more antioxidant activity.

### LC–MS conditions

The active compounds of the extracts and nanoparticles produced of *G. lucidum* were identified by Liquid Chromatography-Mass Spectrometry (LC–MS), based on the method proposed by Yang et al.^34^. The using device in this work was an Agilent (Waldbronn, Germany) 1200 series chromatographic system coupled with an Agilent 6410 triple quadrupole tandem mass spectrometer. LC–MS data were analyzed using the software provided by Agilent Mass Hunter Workstation. An Agilent HT Zorbax SB-C18 column (5 µm particle size, 4.6 mm × 250 mm; Agilent Technologies, Santa Clara, CA) was carried out for the separation process. The mobile phase used in the gradient elution program consisted of acetonitrile and water containing 0.2% acetic acid(v/v) at a flow rate of 1 mL/min, and the injection volume was 5 μL. During 40 min, the volume of acetonitrile increased from 30 to 32% and reached 40% in 20 min. Then it was kept constant at this value for 5 min. The Electron Spray Ionization (ESI) was worked Positive ion mode with the voltage of 4 kV and a capillary temperature equal to 300 °C.

### Investigation of collected nanoparticles

Field emission scanning electron microscopy (FESEM, MIRA3 TESCAN-XMU, and the Czech Republic) was used to characterize the morphology and the size of collected particles. For this purpose, a sputtering system (Pelco SC-7, Ted Pella Inc., Redding, CA) was used to coat a gold layer on the surface of particles. The image analysis was performed using ImageJ software.

## Results

### Optimization of the extraction method

**Figure 2:**
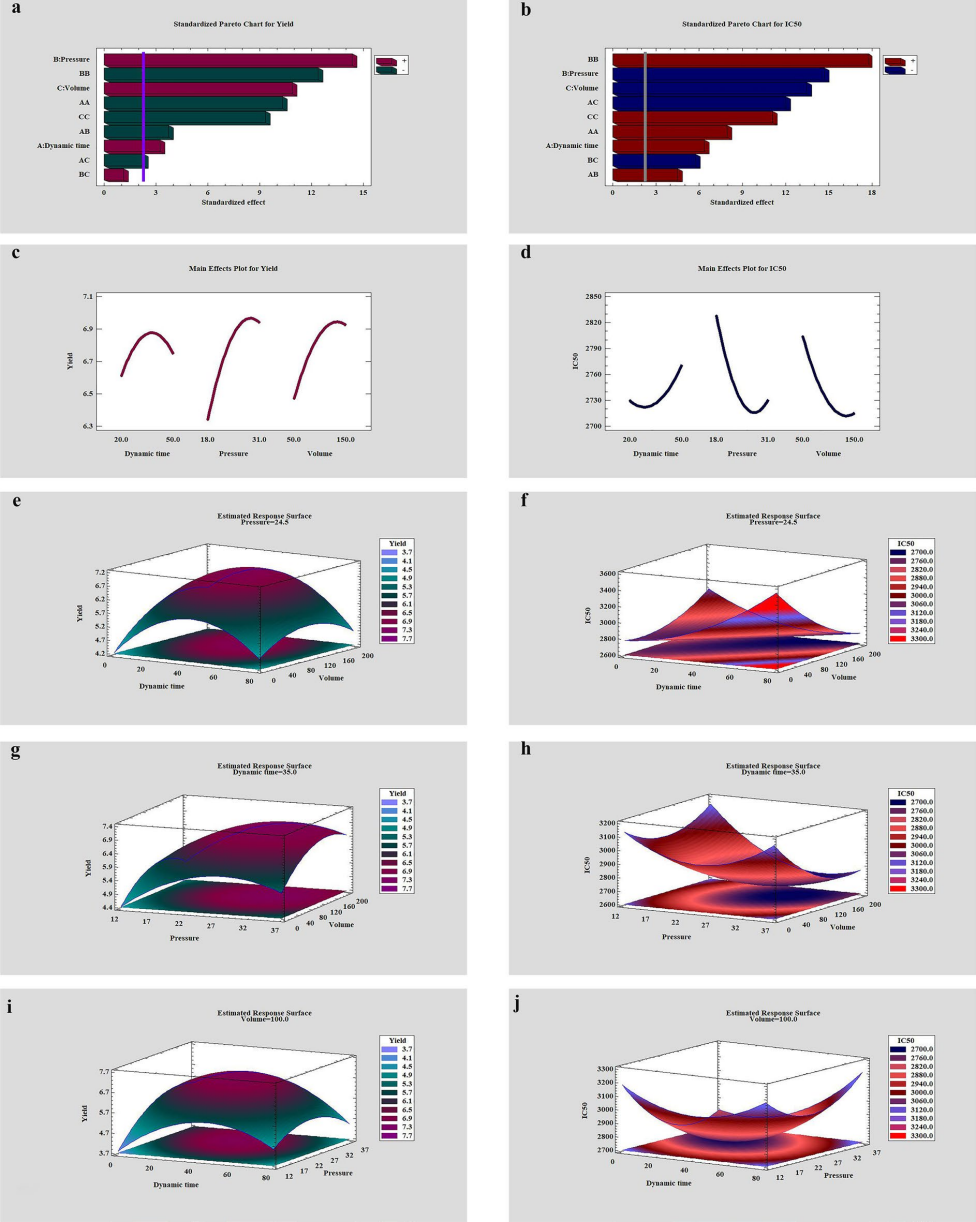
(**a,b**) Standardized Pareto chart for SFE yield and IC_50_, respectively. (**c,d**) Main effect for SFE yield and IC_50_ respectively. Estimated response surface for (**e,f**) temperature was fixed at 55 ˚C, pressure was fixed at 24.5 MPa., (**g,h**) Dynamic time was fixed at 35 min, (**i,j**) modifier volume was fixed at 100 µL for SFE yield and IC_50_ , respectively.

Analysis of variance (ANOVA) was performed to investigate the effect of pressure, temperature, static time, dynamic time, modifier volume on extraction efficiency, and IC_50_ at a 95% confidence level. The half-fractional design presented 16 experiments (2^n−1^, n = 5). Two high and low levels were considered for each variable based on initial experiments, and previous articles^15,35,36^(Table S1). Based on the Pareto chart shown in Figure S1, and results obtained (Table S2), pressure, dynamic time, and modifier volume were selected as effective parameters to find the optimal points in the experiment, and the static time and temperature were kept constant at 10 min and 35 °C respectively. Then the Central Composite Design-cube star (CCD) was optimized, affecting parameters at three levels (Table S3), to obtain the highest extraction efficiency and the lowest IC_50_ value. According to the formula K = k^2^ + 2k + C (Since k is the number of parameters and C is the number of the center points repetition), with 3 center point repetitions in each block, the total number of experiments was achieved 20. The values of the parameters in each experiment, and the results obtained are given in Table 1. The second-order polynomial models were acquired as:

$${\text{Y}}_{{{\text{Yield}}}} = \, - 0.{83913 } + \, 0.0{\text{986991 X}}_{{1}} + \, 0.{34}0{\text{382 X}}_{{2}} + \, 0.0{\text{189977 X}}_{{3}} - \, 0.000{8523}0{\text{7 X}}_{{1}}^{{2}} - \, 0.00{1}0{\text{641 X}}_{{1}} {\text{X}}_{{2}} - \, 0.0000{\text{85 X}}_{{1}} {\text{X}}_{{3}} - \, 0.00{545}0{\text{19 X}}_{{2}}^{{2}} + \, 0.0000{\text{961538 X}}_{{2}} {\text{X}}_{{3}} - \, 0.0000{\text{693745 X}}_{{3}}^{{2}}$$

$${\text{Y}}_{{{\text{IC50}}}}= { 3685}.{64 } - { 3}.{\text{78424 X}}_{{1}} - { 67}.{\text{4843 X}}_{{2}} + \, 0.{\text{895816 X}}_{{3}} + \, 0.{1}0{\text{3761 X}}_{{1}}^{{2}} + \, 0.{2}0{\text{3846 X}}_{{1}} {\text{X}}_{{2}} - \, 0.0{7}0{\text{8333 X}}_{{1}} {\text{X}}_{{3}} + { 1}.{\text{23963 X}}_{{2}}^{{2}} - \, 0.0{78}0{\text{769 X}}_{{2}} {\text{X}}_{{3}} + \, 0.0{13}0{\text{663 X}}_{{3}}^{{2}}$$

where X_1_, X_2_, X_3_ are dynamic time, pressure, and modifier volume, respectively (Table S3). ANOVA’s outcomes showed a good accomplishment R^2^ adjusted and R^2^ of 97.09 and 98.62, for extraction efficiency and ad R^2^ adjusted and R^2^ of 98.24 and 99.68 for IC_50_, respectively. Pareto charts (Fig. 2a,b) related to the experimental design showed that all three parameters, pressure, dynamic time, and modifier volume, affect the extraction efficiency, and the value of IC_50_ at the 95% confidence level. The response surface plots are indicted when the dynamic time was kept constant (35 min, Fig. 2g,h), the interaction of pressure, and volume of modifier has a positive effect on the extraction efficiency and antioxidant activity. The negative correlation between the pressure and the dynamic is shown in Figure (V = 100 µL, Fig. 2i,j). Their increase causes the IC_50_ increment and reduces extraction efficiency. Also, the interaction between modifier volume and dynamic time is negative, as shown in Fig. 2e,f, when the pressure was kept constant at 24.5 MPa. In the following, the effect of the parameters is described.

**Table 1:** Design matrix and the responses for central composite blocked cube-star design.

Experiment	Block	Dynamic time (min)	Pressure (MPa)	Volume (µL)	Yield %	IC_50_ (ppm)
1	1	20	18	150	6.01	2929
2	1	35	24.5	100	6.82	2741
3	1	20	31	150	6.81	2750
4	1	20	18	50	5.43	2866
5	1	50	31	50	6.20	2976
6	1	35	24.5	100	6.82	2743
7	1	50	31	150	6.65	2725
8	1	35	24.5	100	6.81	2734
9	1	50	18	50	5.94	2974
10	1	20	31	50	6.23	2785
11	1	50	18	150	6.14	2828
12	2	35	12.7	100	5.70	2973
13	2	35	24.5	100	6.77	2739
14	2	35	24.5	9.5	6.00	2890
15	2	35	24.5	190	6.81	2739
16	2	35	24.5	100	7.02	2701
17	2	35	24.5	100	7.00	2702
18	2	8	24.5	100	6.20	2748
19	2	62	24.5	100	6.49	2820
20	2	35	36	100	6.80	2785

#### Pressure effect

The solubility of the solute in the supercritical fluid directly affects the extraction performance.

Increasing the pressure, increases the density, and consequently increases the contact surface between the extractable compounds and the extraction solvent^37,38^. Main effect charts (Fig. 2c,d) indicate the positive effect of pressure on extraction efficiency and antioxidant properties (adverse effect on IC_50_). In addition, increasing the pressure reduces the penetration of the fluid into the sample matrix, which reduces the mass transfer rate. The reverse pressure behavior at high values can be attributed to this effect.

#### Modifier solvent volume effect

Increasing the modifier solvent volume increases the extraction efficiency, and decreases the amount of IC_50_, and in the result is increased antioxidant activity (Fig. 2c,d). This is because, increasing the volume of the modifier solvent improves the extraction of polar compounds^15^. Of course, an excessive increase in the solvent volume of the modifier interferes with the extraction of non-polar compounds.

#### Dynamic time effect

The interaction of compounds with supercritical fluid increases with enhancing dynamic time, and fluid can carry more compounds. As a result, the extraction efficiency and, consequently the antioxidant properties increase. However, it should be noted that as the dynamic time increases, as the fluid separates from the compounds during sample collection, some of the compounds are released with the fluid, which reduces the extraction efficiency^39^.

### Optimization of the nanoparticles production process

**Figure 3:**
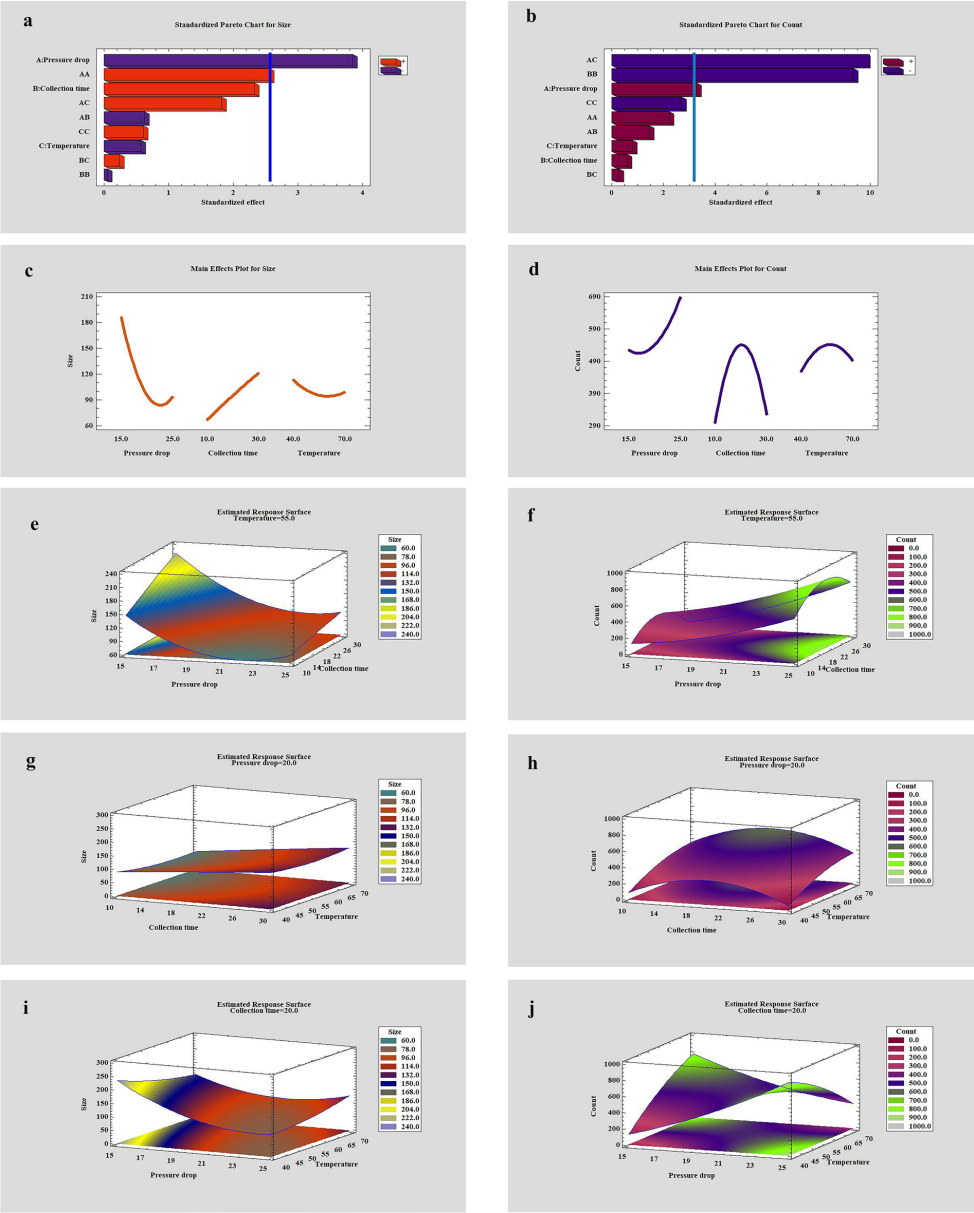
(**a,b**) Standardized Pareto chart for size and count respectively. (**c,d**) Main effect for size and count respectively. Estimated response surface for (**e,f**) temperature was fixed at 55 ˚C, (**g,h**) pressure was fixed at 20 MPa, (**i,j**) collection time was fixed at 20 min for size and count respectively.

The selected values for the parameters of pressure, temperature, equilibrium time, collection time, the volume of the extracted solution were based on the initial experimental results and the information obtained from previous studies. Accordingly, the volume of the extracted solution was measured in three values of 30, 40, and 50 µl. The results showed that after completing the process in volumes of 40 and 50 µl, the solvent remains inside the capsule. Then a volume of 30 μL was used for all stages of the experiment. Also, according to previous studies, the value of equilibrium time in all experiments was kept constant for 30 minutes^27,28^. To find the optimal point for the highest number of formed nanoparticles with the smallest size, the parameters of pressure drop, collection time, and temperature were examined by Statgraphics software. Experimental design, Box Behnken was used to optimizing the parameters in three levels. The experimental conditions and effective parameters in the production of nanoparticles are presented in Table 2. The following second-order polynomial models were acquired as:

$${\text{Y}}_{{{\text{Size}}}} = { 1491}.{7 } - { 98}.{6} \times {\text{A }} + { 5}.{85} \times {\text{B }} - { 14}.{29}0{7} \times {\text{C }} + { 1}.{78333} \times {\text{A}}^{{2}} - \, 0.{21} \times {\text{A}} \times {\text{B }} + \, 0.{4}0{6667} \times {\text{A}} \times {\text{C }} - \, 0.00{916667} \times {\text{B}}^{{2}} + \, 0.0{266667} \times {\text{B}} \times {\text{C }} + \, 0.0{47}0{37} \times {\text{C}}^{{2}}$$

$${\text{Y}}_{{{\text{Count}}}} = \, - {4822}.{98 } + { 123}.0{72} \times {\text{A }} + { 72}.{425} \times {\text{B }} + { 116}.{498} \times {\text{C }} + { 2}.{64167} \times {\text{A}}^{{2}} + \, 0.{921667} \times {\text{A}} \times {\text{B }} - { 4}.{19222} \times {\text{A}} \times {\text{C}} - { 2}.{29625} \times {\text{B}}^{{2}} + \, 0.0{383333} \times {\text{B}} \times {\text{C}} - \, 0.{29463} \times {\text{C}}^{{2}}$$

where A, B, C are pressure drop, collection time, and temperature, respectively (Table S4). Based on the values obtained for R^2^, the quadratic polynomial model obtained is sufficient to describe the experimental results, (86.13, and 98, for size and count, respectively). The optimum point for the production of nanoparticles was obtained as follows: pressure drop at 25 MPa, 20 min for collection time, and 40° C for temperature. Also, based on the Pareto charts (Fig. 3a,b) and, the surface response diagrams that show the interaction of two-by-two parameters (Fig. 3e–j), only the interaction of pressure drop and the temperature has a significant effect (negative) on the number of particles.

**Table 2:** Design matrix and the responses (BBD) for the production of nanoparticles.

Experiment	Pressure drop (MPa)	Collection time (min)	Temperature (˚C)	Size (nm)	Count	Figures
1	25	30	55	141	1404	–
2	15	20	40	264	101	4a
3	20	30	70	130	250	4b
4	20	20	55	95	540	4c
5	15	30	55	222	286	–
6	25	20	40	81	1021	4d
7	20	30	40	105	235	–
8	20	20	55	99	561	–
9	15	20	70	159	787	–
10	25	20	70	98	351	–
11	15	10	55	116	329	4e
12	25	10	55	77	376	–
13	20	10	40	88	251	–
14	20	10	70	97	243	–
15	20	20	55	92	521	4f

#### Pressure drop effect

In this study, the pressure drop was investigated in the range of 15–25 MPa. So that the secondary pressure was varied in selected values by the software (above the supercritical pressure of carbon dioxide), and the primary pressure was kept constant at 35 MPa.

**Figure 4:**
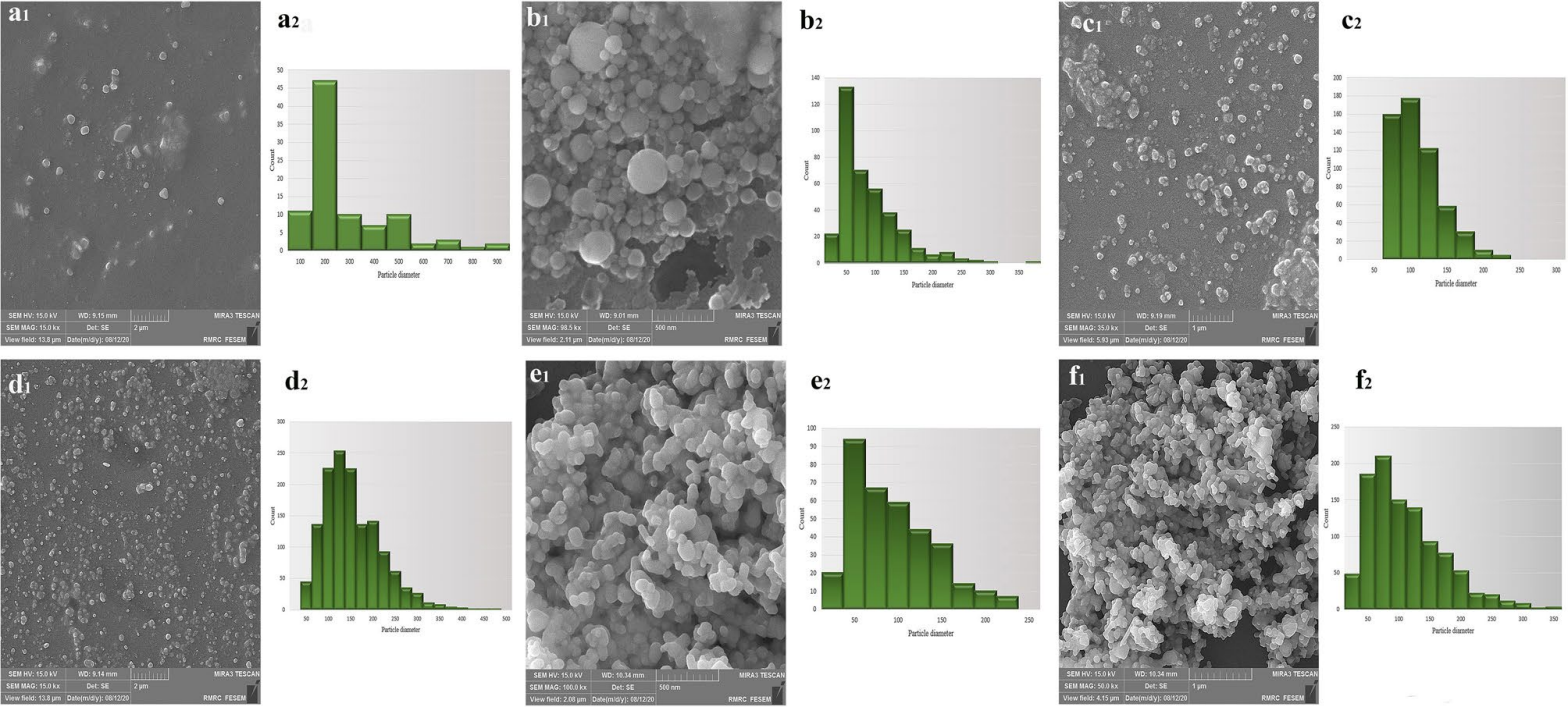
FESEM images of produced nanoparticles: (**a**_**1**_**–f**_**1**_) experiments 2, 3, 4, 6, 11 and 15 respectively. Corresponding particle size diagram: (**a**_**2**_**–f**_**2**_) experiments 2, 3, 4, 6, 11 and 15 respectively.

Pareto and main effect diagrams (Fig. 3a–d) showed that the pressure drop had the most significant effect on the size and number of formed nanoparticles. As the pressure drop decreases, the size of the particles formed decreases, and their number increases. To explain this phenomenon, it can be said that with increasing pressure drop, the density of SC-CO_2_ and the collision between CO_2_ molecules and the solute increases. According to the nucleation theory, increasing the solubility of the solute in CO_2_ increases the relative supersaturation in the solution and decreases the particle size. More nucleation causes more particles to be produced^12,40^. This trend is observed until it reaches the optimal point, and then a reverse trend is observed due to the accumulation of particles. The effect of pressure on the formation of nanoparticles and related diagrams (experiments 2 and 3) is shown in Fig. 4a1,a2,b1,b2.

#### Temperature effect

In this study, the temperature was examined in the range of 40–70 °C. The temperature has two different effects on the nanoparticle formation process; As the temperature increases, the CO_2_ density decreases, causing the solubility of the supercritical fluid to decrease. In addition, increasing the temperature increases the vapor pressure of the solute in SC-CO_2_, which increases the solubility of the supercritical fluid. In this study, with increasing temperature, the particle size decreases and their number increases, and then the reverse process is observed. Figures and diagrams Fig. 4d1,d2 (experiment 6) and Fig. 4f_1_,f_2_ (experiment 15) show the effect of temperature on the produced nanoparticles.

#### Collection time effect

As the collection time increases, the number of particles formed increases to some extent, and then a decreasing trend is observed. Particle size also increases over time, which can be due to particle accumulation. Figures and diagrams Fig. 4c1,c2 (experiment 4) and Fig. 4e_1_,e_2_ (experiment 11) show the effect of temperature on the produced nanoparticles.

#### Evaluation of antioxidant properties of nanoparticles

The antioxidant properties of nanoparticles produced at the optimum point were evaluated by the DPPH method and compared with the obtained IC_50_ value at the optimum point of extraction efficiency. The IC_50_ results for the produced nanoparticles and the extract obtained under optimal conditions were 580 and 724 ppm, respectively. These values indicate that as the particle size decreases, the antioxidant activity increases.

### Identification of compounds

**Figure 5:**
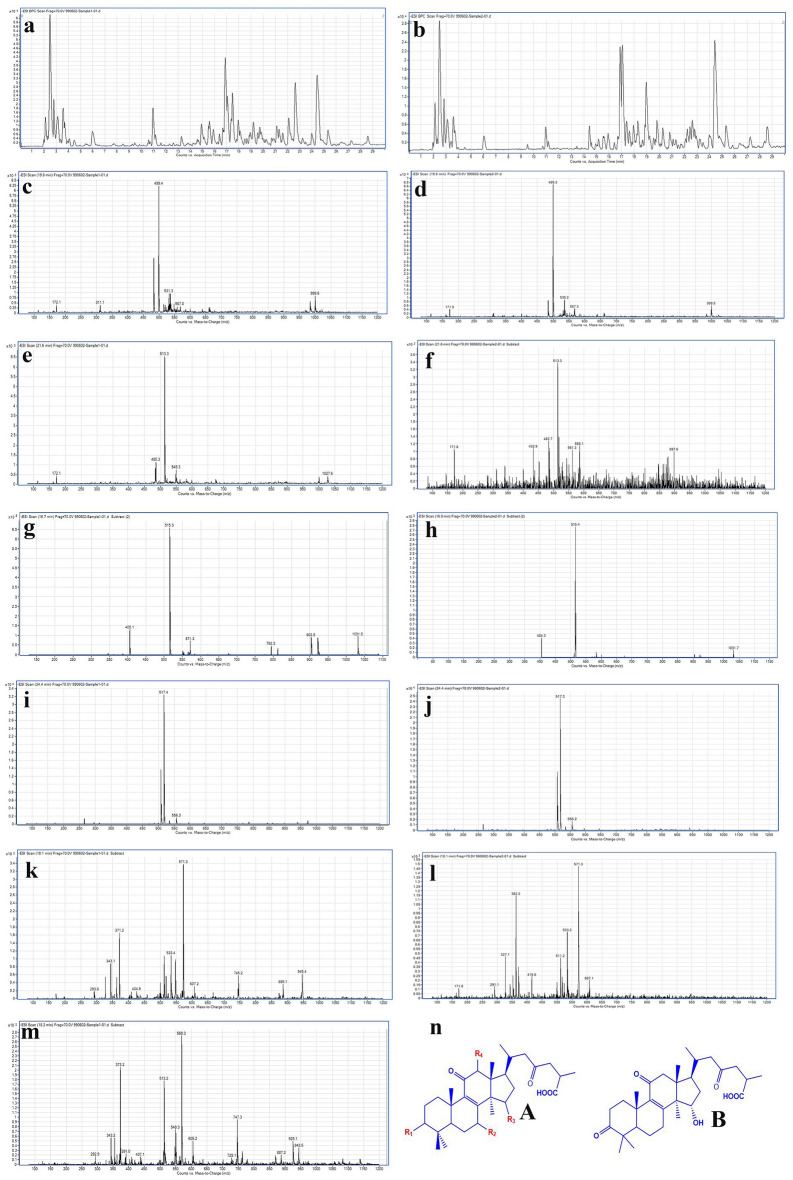
(**a,b**) The extracted ion chromatogram(EIC) of herbal extract and nanoparticles, respectively. (**c,d**) Ganolucidic acid mass spectrum, related to the herbal extract and nanoparticles, respectively. (**e,f**) Ganoderic acid D mass spectrum, related to the herbal extract and nanoparticles, respectively. (**g,h**) Ganoderic acid B mass spectrum, related to the herbal extract and nanoparticles, respectively. (**i,j**) Ganoderic acid C_2_ mass spectrum, related to the herbal extract and nanoparticles, respectively. (**k,l**) Ganoderic acid H mass spectrum, related to the herbal extract and nanoparticles, respectively. (**m**) 12-acetoxy ganoderic acid F mass spectrum, related to the herbal extract. (**n**) The structure of the identified compounds.

*G. lucidum* has numerous compounds, including a group of triterpenes called ganoderic acids. Identification of compounds was performed by LC–MS to identify triterpenes in extracts and nanoparticles based on the method proposed by Yang et al.^34^. Based on their observations, the negative ion signals [M − H]^−^ triterpenes were well detected, but the positive ion signals were measurable with less intensity than [M − H]^−^. Therefore, the negative ion state was chosen for measurement, and in the present work, the negative ion state was performed. The substances identified in the extracts and nanoparticles are presented in Table 3 and Fig. 5.

**Table 3:** Compounds identified in nanoparticles and extracts of Ganoderma by LC–MS.

Compound	MW	Type	R_1_	R_2_	R_3_	R_4_	RT	Herbal extract	Nanoparticle
Ganolucidic acid	500	B	–	–	–	–	19.8	Figure 5c	Figure 5d
Ganoderic acid D	514	A	=O	β-OH	=O	H	21.6	Figure 5e	Figure 5f
Ganoderic acid B	516	A	β-OH	β-OH	=O	H	16.7	Figure 5g	Figure 5h
Ganoderic acid C_2_	518	A	α-OH	β-OH	β-OH	H	24.1	Figure 5i	Figure 5j
Ganoderic acid H	572	A	β-OH	=O	=O	β-OAc	18.1	Figure 5k	Figure 5l
12-Acetoxy ganoderic acid F	570	A	=O	=O	=O	β-OAc	18.2	Figure 5m	–

R_1_, R_2_, R_3_ ,R_4_ are functional groups that are replaced in Fig. 5n.

## Conclusion

The fungus was used several thousand years old due to its nutritional and medicinal value. *G. lucidum* is a type of fungus with unique medicinal properties, which was introduced by East Asian countries. *G. lucidum* contains compounds such as polysaccharides and triterpenoids used in the treatment of various diseases such as cancer, hypotension, detoxification, etc. A large part of the triterpenoids in this plant is ganoderic acids. This study was performed to identify the ganoderic acids of *G. lucidum* and to produce nanoparticles using supercritical fluid expansion. *G. lucidum* was extracted after crushing, grinding, and passing through standard sieves, and its antioxidant properties were evaluated. The obtained extract was used to produce nanoparticles at the optimum point. Optimal conditions were observed at the extraction step to get maximum efficiency and antioxidant activity at a pressure of 27.5 MPa, dynamic time of 46 min, and modifier volume of 162 μL, while according to the results of the screening step, temperature and static time were kept constant at 45 °C and 10 min, respectively. Under these conditions, the extraction efficiency and IC_50_ were 7.1 and 764.2 ppm, respectively. Also, based on the results, it was observed that the pressure parameter, modifier volume and dynamic time have the greatest effect on IC_50_ value and extraction efficiency, respectively. Optimal conditions in the nanoparticle production step to produce the largest number of particles formed with the smallest size, including pre-expansion pressure of 35 MPa, post-expansion pressure of 10 MPa, the temperature of 40 °C, collection time of 20 min, equilibrium time of 30 min and volume of solution extraction of 30 µL. Under these conditions, the size and count were 86.13 nm, and 98, respectively. In the nanoparticle production process, it was observed that the pressure drop parameter has a greater effect on the number and size of nanoparticles produced. Herbal extracts and produced nanoparticles were identified by LC–MS that were included Ganoderic acid B, Ganoderic acid C_2_, Ganoderic acid D, Ganoderic acid H, and Ganolocidic acids. It was also shown using the DPPH method that the antioxidant properties of the nanoparticles have been improved. The IC_50_ results for the produced nanoparticles and the extract obtained under optimal conditions were 580 and 724 ppm, respectively. Based on the results of previous studies and the data obtained, which show the optimum points obtained for each in table S5, increasing the pressure drop had the greatest effect on the production of particles with the smallest size. Thus, the ESS process is a beneficial approach to produce nanoparticles, and it can enhance the bioavailability of insoluble or poorly soluble phytochemical compounds of herbal medicine.

## Supplementary Information

Supplementary Information.

## Abbreviations

*G. lucidum*: *Ganoderma lucidum*
ESS: Expansion of supercritical fluid solution
FESEM: Field emission scanning electron microscopy
LC–MS: Liquid chromatography–mass spectrometry
DPPH: 2,2-Diphenyl-1-picrylhydrazyl
SC-CO_2_: Carbon dioxide supercritical fluid
RESS: Rapid expansion of supercritical solution
SAS: Supercritical anti-solvent
PGSS: Particles from gas-saturated solution
DMSO: Dimethyl sulfoxide
SFE: Supercritical fluid extraction
UV–Vis: Ultraviolet–visible spectroscopy
ESI: Electron spray ionization
ANOVA: Analysis of variance
CCD: Central composite design-cube star
