# Supplementary material for: Production Ganoderma lucidum extract nanoparticles by expansion of supercritical fluid solution and evaluation of the antioxidant ability
Source: Sci Rep. 2022 Jun 14;12:9904. doi: 10.1038/s41598-022-13727-8 (PMC9198024; doi:10.1038/s41598-022-13727-8)
Supplement: Supplementary file 1 — Supplementary Information. [file 41598_2022_13727_MOESM1_ESM.docx]

*For submission to The Nature Scientific Reports, October 2021.*

Production Ganoderma lucidum extract nanoparticles by Expansion of Supercritical Fluid Solution and evaluation of the antioxidant ability

Mehrnaz Karimi, Farhad Raofie***^1^, Mehrdad Karimi^2^

1. *Department of Analytical Chemistry and pollutants, Shahid Beheshti University, Tehran*

*, Iran, 1983969411*

1. *Department of Traditional Medicine, School of Persian Medicine, Tehran University of Medical Sciences, Tehran, Iran, 1417613151.*

** Corresponding author, email: f_raofie@sbu.ac.ir Fax: +98-21-22431661*

**Supplementary material**

**Figure:**

**
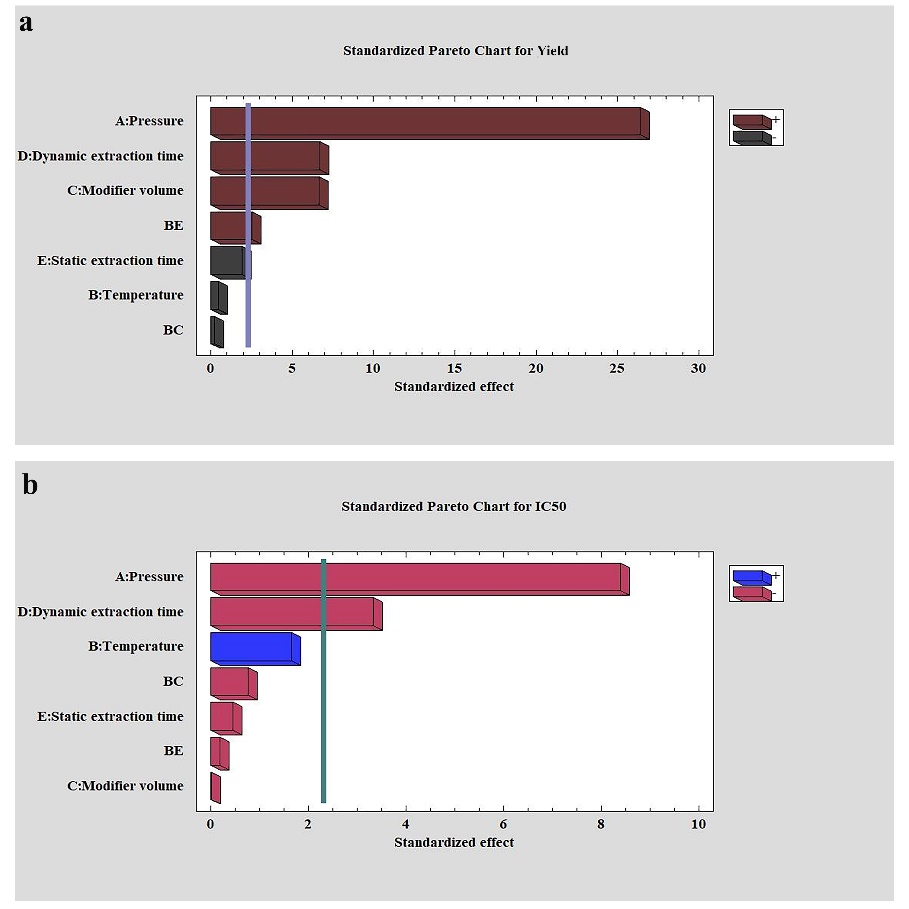
**

Figure S1: (a), (b) Standardized Pareto chart for SFE yield and IC_50_, respectively in the screening step.


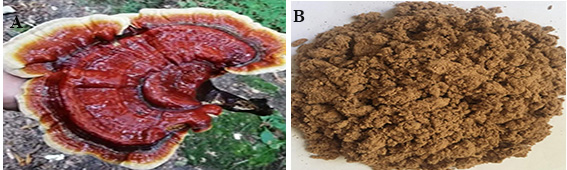


Figure S2: a) Ganoderma lucidum; b) powdered sample

**Tables**

**Table S 1 :** High and low levels selected for half-fraction design in the screening step.

| **Levels** | **Pressure** | **Temperature** | **Modifier**  **volume** | **Static**  **Time** | **Dynamic**  **time** |
| --- | --- | --- | --- | --- | --- |
|  | **(MPa)** | **(ºC)** | **(µL)** | **(min)** | **(min)** |
| **Low** | 15 | 35 | 0 | 10 | 10 |
| **High** | 35 | 65 | 100 | 30 | 45 |

**Table S2 :** Design matrix and the responses for Half-Fraction design for Screening.

| **Experiment** | **Pressure** | **Temperature** | **Modifier volume** | **Dynamic time** | **Static time** | **Yield** | **IC_50_** |
| --- | --- | --- | --- | --- | --- | --- | --- |
|  | MPa | C | µL | min | min | % |  |
| **1** | 35 | 65 | 100 | 10 | 10 | 6.30 | 938 |
| **2** | 35 | 65 | 0 | 10 | 30 | 6.10 | 995 |
| **3** | 15 | 65 | 0 | 10 | 10 | 5.23 | 1210 |
| **4** | 15 | 65 | 0 | 45 | 30 | 5.42 | 1113 |
| **5** | 35 | 35 | 0 | 45 | 30 | 6.21 | 763 |
| **6** | 15 | 35 | 100 | 45 | 30 | 5.59 | 1038 |
| **7** | 35 | 35 | 100 | 10 | 30 | 6.21 | 984 |
| **8** | 15 | 35 | 0 | 45 | 10 | 5.50 | 1075 |
| **9** | 35 | 65 | 0 | 45 | 10 | 6.15 | 874 |
| **10** | 35 | 35 | 100 | 45 | 10 | 6.62 | 901 |
| **11** | 15 | 35 | 100 | 10 | 10 | 5.43 | 1087 |
| **12** | 15 | 65 | 100 | 45 | 10 | 5.63 | 1162 |
| **13** | 35 | 65 | 100 | 45 | 30 | 6.54 | 831 |
| **14** | 35 | 35 | 0 | 10 | 10 | 6.16 | 920 |
| **15** | 15 | 65 | 100 | 10 | 30 | 5.31 | 1176 |
| **16** | 15 | 35 | 0 | 10 | 30 | 5.09 | 1170 |

**Table S 3 :** The experimental variables and levels Central Composite Design cube-star (CCD).

| **Studied variables** | | **Variable level** | | |
| --- | --- | --- | --- | --- |
|  |  | High | medium | low |
| **X_1_** | Dynamic time (min) | 50 | 35 | 20 |
| **X_2_** | Pressure (MPa) | 31 | 24.5 | 18 |
| **X_3_** | Modifier volume (µL) | 150 | 100 | 50 |
| **Response** |  | **Aim** | | |
| **Y_Yield_** | Extraction efficiency (%) | Maximum | | |
| **Y_IC50_** | Antioxidant activity (ppm) | Minimum | | |

**Table S4:** The experimental variables and levels Box-Behnken Design(BBD).

| **Studied variables** | | **Variable level** | | |
| --- | --- | --- | --- | --- |
|  |  | High | medium | low |
| **A** | Pressure drop (MPa) | 50 | 35 | 20 |
| **B** | Collection time (min) | 31 | 24.5 | 18 |
| **C** | Temperature (˚C) | 150 | 100 | 50 |
| **Response** |  | **Aim** | | |
| Y _Particle Size_ | Particle size (nm) | Minimum | | |
| Y _Count_ | Count | Maximum | | |

**Table S5:** statistically optimized condition of previous studies

| **Plant** | **Identified Compounds** | **Parameters Condition** | | | | | **Size**  **nm** | | **Count** | **Reference** |
| --- | --- | --- | --- | --- | --- | --- | --- | --- | --- | --- |
|  |  | **dp** | **T** | **t _1_** | **t _2_** | **V** |  |  |  |  |
|  |  | **Mpa** | **ºC** | **min** | **min** | **µL** |  |  |  |  |
| Catharanthus roseus | Vincristine | 23.3 | 50 | 10 | 30 | 30 | 5-200 | | - | ^1^ |
|  |  |  |  |  |  |  |  | |  |  |
| Peganum harmala | B-carboline alkaloids | 25.3 | 40 | 10 | 10 | 50 | 7-100 | | - | ^2^ |
|  |  |  |  |  |  |  |  | |  |  |
| Withania somnifera | Withaferin A | 25.7 | 53 | 23 | 57 | 30 | 5 | 5842 | | ^3^ |
| Citrus sinensis | bioflavanids | 25.3 | 40 | 10 | 10 | 50 | 5-100 | | - | ^4^ |
| silybum marianum | Silymarin derivatives | 23.3 | 55-60 | 30 | 60 | 20 | 71-74 | | 300 | ^5^ |
| Curcuma Longa | Curcumin derivatives | 11 | 50 | 30 | 60 | 30 | 47 ± 20 | | 300 | ^6^ |
| Ganoderma lucidum | Ganoderic acids | 25 | 40 | 30 | 20 | 30 | 86 | | 98 |  |

dp: pressure drop; T: temperature; t _1_: equilibrium time; t _2_ : collection time; V: volume of the extract solution.
